# Prenatal Care in Combination with Maternal Educational Level Has a Synergetic Effect on the Risk of Neonatal Low Birth Weight: New Findings in a Retrospective Cohort Study in Kunshan City, China

**DOI:** 10.1371/journal.pone.0113377

**Published:** 2014-11-26

**Authors:** Lin-Lin Dai, Yuan-Yuan Mao, Xiao-Ming Luo, Yue-Ping Shen

**Affiliations:** 1 Department of Epidemiology and Biostatistics, School of Public Health, Medical College of Soochow University, Suzhou, Jiangsu, China; 2 Kunshan first people’s Hospital, Kunshan, Suzhou, Jiangsu, China; 3 Kunshan fourth people’s Hospital, Kunshan, Suzhou, Jiangsu, China; Iran University of Medical Sciences, Iran, Islamic Republic Of

## Abstract

**Objectives:**

To investigate the dose-response relationship and synergetic effect of the maternal educational level and two measures of prenatal care on neonatal low birth weight (LBW) risk.

**Methods:**

Data were derived from the Perinatal Health Care Surveillance System (PHCSS) from January 2001 to September 2009 in Kunshan City, Jiangsu province, eastern China, which included data on 31412 women with a normal birth weight delivery and 640 women with a LBW delivery. Logistic modelling was performed to estimate the association including the joint effects with odds ratio (OR) and 95% confidence interval (CI) between the prenatal care measures and LBW risk after adjusting for the potential confounders. The dose-response relationship between the number of prenatal care visits and the risk of LBW was investigated by modeling the quantitative exposure with restricted cubic splines (RCS).

**Results:**

There was a significant synergetic effect on the LBW risk between maternal educational attainment and the number of prenatal care visits (χ^2^ = 4.98, *P* = 0.0257), whereas no significant maternal educational attainment interaction was found with the week of initiation of prenatal care after adjusting for relevant confounding factors (χ^2^ = 2.04, *P* = 0.1530), and the LBW risk displayed a ‘U-shape’ curve tendency among the different number of prenatal care visits (*P* for nonlinearity = 0.0002) using RCS. In particular, the ORs were approaching the curve’s bottom when the women had 9 or 10 prenatal care visits. Comparing with 5 prenatal care visits, the ORs and 95%CI of LBW risk for 7, 9, 11 and ≥13 visits were 0.92 (0.82–1.03), 0.50 (0.38–0.66), 0.62 (0.47–0.82), and 0.99 (0.61–1.60), respectively.

**Conclusions:**

Our findings suggest that appropriate prenatal care, in combination with a higher maternal educational level, can produce a protective interaction effect on LBW risk. Reasonable health resource assignment for different social statuses should be taken into account by policy-makers in developing countries.

## Introduction

Low birth weight (LBW), a common adverse obstetrical outcome, has been reported extensively as a significant contributor to perinatal morbidity and mortality as well as unhealthy outcomes later in life, including respiratory distress syndrome, heart disease and diabetes mellitus [1]–[4]. In the last few decades, prenatal care has been generally recognized as an effective method of preventing LBW by imparting of information, screening for abnormalities and providing timely intervention [5]–[8]. However, some studies have demonstrated that prenatal care has failed to reduce the occurrence of poor outcomes. From the early 1980 s to 2007, the rate of LBW failed to decrease in the USA despite increased prenatal care utilization from Medicaid coverage for prenatal care services aimed at a reduction in LBW [9]. This phenomenon was also found in Brazil during the 15-year period of 1979–1994 [10]. In addition, a 2003 review reported that the present prenatal care could prevent neither preterm birth nor intrauterine growth retardation effectively, which were the major causes of LBW [11].

Epidemiological evidence suggests that many socioeconomic factors, such as race, maternal country of birth, family income and occupation, affect the provision and uptake of prenatal care in both developed and developing countries [12]–[15]. A recent Chinese study examined three separate socioeconomic indices (wealth, occupation and education) related to perinatal care and outcomes obtained from a principal component analysis for rural Chinese pregnant women [16]. It was found that not only did a low socioeconomic level lead to a lack of prenatal care utilization, but it also produced varied unsatisfactory pregnancy courses, and this socioeconomic unfairness was expanding [17]. A study of 6,159,070 full-term, live-born singletons in Taiwan revealed the LBW risk ratios of the lowest-educated mothers to the highest-educated mothers increased from 1.43 in 1978–1979 to 2.05 in 1996–1997 [18]. Another similar study also concluded education-related inequalities associated with LBW persisted from the early to the late 1990 s in 6 counties in southern China [19].

Most studies explain the relationship between prenatal care and pregnancy outcomes using categorical prenatal care indices such as the Kessner index, GINDEX, adequacy of prenatal care utilization (APNCU) and certain variants of the APNCU, which divide prenatal care utilization into 3 or 4 categories [8], [20]. A few studies focus attention on the number of antenatal visits to demonstrate a dose-response relationship [5]. In China, most of the relevant studies have focused on whether the total number of prenatal care visits reached the recommended value (4 or 5 times) and whether the week of initiation of prenatal care was ≤12, which are somewhat rough criteria [21]. Furthermore, the inequity in prenatal care utilization among Chinese women has not been improved although people’s living standards have greatly improved in recent decades [19].

To our knowledge, there is no published study to date that has evaluated the dose-response relationship between prenatal care and the risk of LBW using the flexible restricted cubic spline model. In addition, the interaction effect between prenatal care and socioeconomic factors has not been estimated although plentiful articles indicate that both of them affect the risk of LBW. We intended in this study to explore the dose-response effect of the number of prenatal care visits and the week of initiation of prenatal care on the risk of LBW in Kunshan City, China, from January 2001 to September 2009. We further evaluated whether maternal educational level, as one of socioeconomic factors, had a joint association with prenatal care on the risk of LBW.

## Materials and Methods

### Data Sources

**Figure 1:**
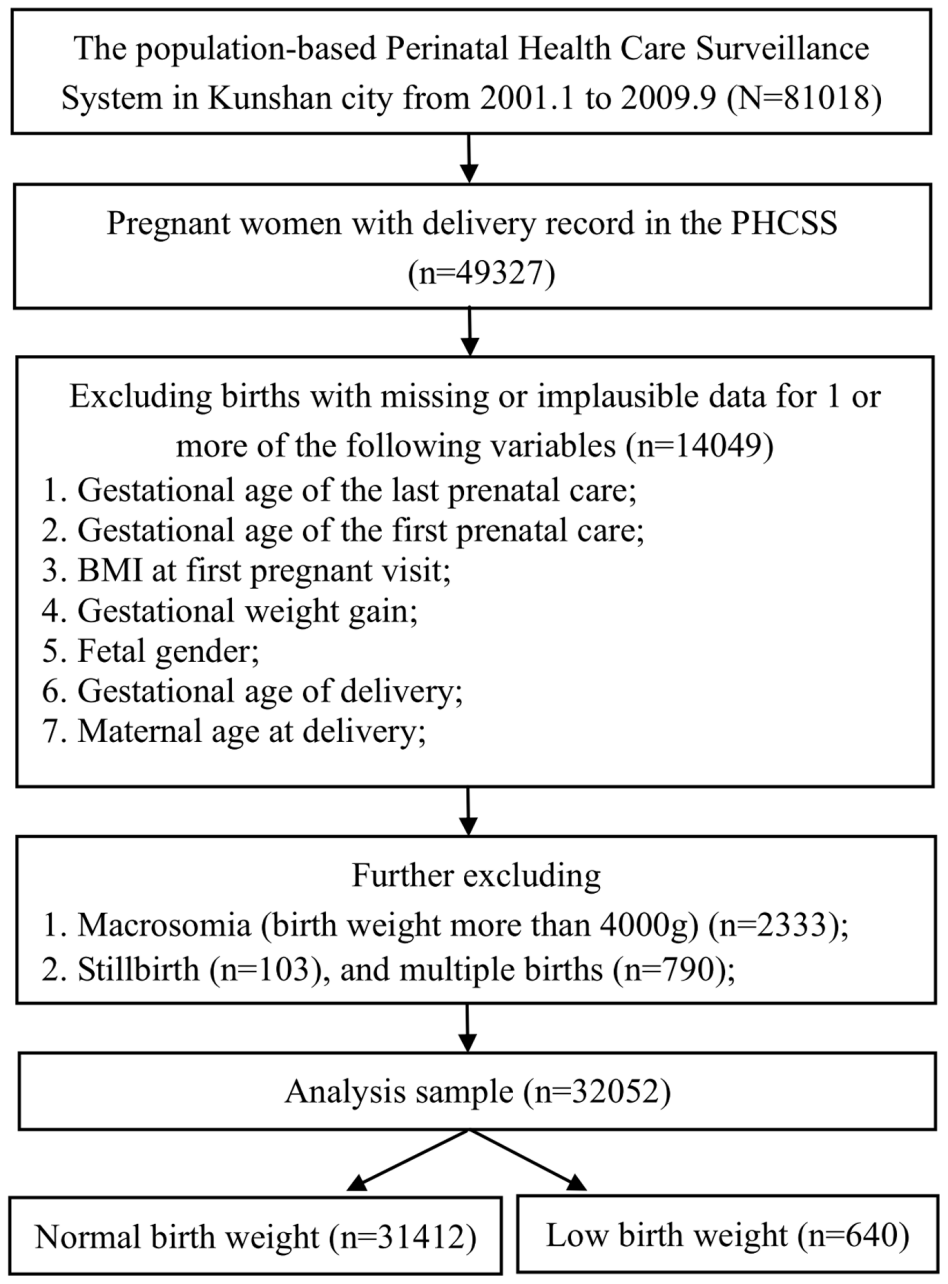
Flow chart.

The study was based on Perinatal Health Care Surveillance System (PHCSS) from January 2001 to September 2009 collected in Kunshan City, Jiangsu province, eastern China, which enrolled all women who were planning to be married or become pregnant and then followed them throughout pregnancy. The information collected through this system includes maternal demographic characteristics, preconception health status, health care services utilization, perinatal health status, pregnancy outcome, and postpartum health status of the mother and infant. We excluded the women with missing or implausible data for these parameters (n = 14049): gestational age of the last prenatal care, gestational age of the first prenatal care, BMI at the first pregnancy visit, gestational weight gain, fetal gender, gestational age of delivery, and maternal age at delivery. In the following analyses, infants with a birth weight more than 4000 g (n = 2333), stillbirth (n = 103), and multiple births (n = 790) were excluded as well. Macrosomia were excluded as an adverse obstetrical outcome, which made it appropriate to assess the risk of prenatal care on LBW compared with normal weight infants (2500–3999 g). Multiple births were excluded because they are at higher risk for low birth weight compared to singleton births. The final study sample was limited to live-born singleton infants including 31412 normal birth weight infants and 640 LBW infants as illustrated in the flow chart in Figure 1. The study was reviewed by Soochow University Institutional Review Board and was considered as exempt from full review as the study was based on an anonymous data set with no identifiable information on the survey participants.

### Definitions

Birth weight was recorded in the hospital record in the first hour after birth and entered into the PHCSS no later than 2 days after birth. LBW, low birth weight, was defined as a birth weight less than 2500 g. In our study, a pregnancy visit was defined as a woman seeking counseling from a health care professional and receiving relevant medical examinations, including physical examination, type-B ultrasound, electrocardiography, etc. in a hospital. The number of prenatal care visits acted as a proxy measure of the overall quantity of pregnancy care received by women. The week of initiation of prenatal care was the gestational week when a woman attended her first pregnancy visit. Gestational weight gain was defined as the average increase of weight from the first pregnancy visit to the last visit per gestational week. The value was calculated by dividing the increase of weight from the first pregnancy visit to the last pregnancy visit by the difference of the gestational week of this period. In this retrospective cohort study, women who had ≤5 prenatal care visits during the pregnancy or initiated prenatal care before 9th week were defined as the non-exposed group, while the others were the exposed groups. And women delivering an LBW was considered as the follow-up outcome.

### Statistical analyses

Continuous data are described as the mean ± standard deviation (SD) or median (P_25_–P_75_), and categorical data are described using proportions. Continuous variables in different groups of the number of prenatal care visits or the week of initiation of prenatal care were compared using analyses of variance or the rank sum test. The chi-square test was used to compare the difference for the proportion or prevalence between different groups.

The number of prenatal care visits and the week of initiation of prenatal care were categorized into four groups according to the quartile distribution of pregnant women delivering normal birth weight newborns. We first examined the associations between the number of prenatal care visits, the week of initiation of prenatal care, maternal educational attainment and the risk of LBW from a simple logistic regression. And then we adjusted for the potential confounders in the multiple logistic regression model, including maternal age, gestational weight gain, BMI at the first pregnancy visit, gestational age, neonatal sex, pregnancy-induced hypertension, maternal job, and prenatal care institutions. The week of initiation of prenatal care, the number of prenatal care visits and maternal educational attainment were adjusted mutually. Crude and adjusted odds ratio (OR) with 95% confidence interval (95% CI) for each quartile were reported from SAS output. An OR was considered statistically significant if its 95% CI did not include 1.0.

Furthermore, to assess the joint effect between the number of prenatal care visits and the maternal educational attainment compared with the reference group on the LBW risk, we produced 12 dummy variables through combining of four-level of prenatal care visits (≤5, 6–8, 9–10 and>10) and three-level of maternal educational attainment (junior middle school or below, senior middle school and junior college degree or above). Then we re-constructed a new logistic model through introducing 11 dummy variables (put the women with both ≤5 prenatal care visits and junior middle school or below educational level as the reference group) and other potential confounders. In order to test the interaction effect statistically, we simultaneously drew the product-term: 4-level maternal educational variable * 3-level numbers of prenatal care visits variable into the multiple logistic model [22]. The analyses of the week of initiation of prenatal care was the same as the number of prenatal care visits.

Taking into account a continuous exposure in regression models by using categorization, when non-linear dose-response associations are expected, have been widely criticized for several limitations including loss of information and reduction in power. And restricted cubic spline functions are powerful tools to characterize a dose-response association between a continuous exposure and an outcome. So we explored the dose-response relationship between the number of prenatal care visits and the risk of LBW by modeling the quantitative exposure with restricted cubic splines with 4 knots at the 20th, 40th, 60th, and 80th percentiles of the distribution [23]. ORs and 95% CI of different prenatal care visits in comparison with the 5 prenatal visits which was recommended in China [21] were shown in the figure in the form of curve. The coefficients of the second spline transformation equal to zero were tested to obtain probability values for nonlinearity.

P<0.05 was considered statistically significant. Statistical analyses were conducted using SAS Version 9.2, and the analysis of the dose-response relationship was conducted using Stata Version 11.2.

## Results

The baseline characteristics of pregnant women and their infants according to the number of prenatal care visits are shown in Table 1. The number of prenatal care visits decreased with the increasing of maternal age, BMI at the first pregnancy visit and gestational weight gain. Moreover, the number increased if pregnant women had earlier initiation of prenatal care or larger gestational age at delivery. Male sex, maternal job as a farmer, a maternal educational level of junior middle school or below, and receiving prenatal care in general hospitals were associated with fewer prenatal care visits. There was no difference between women with pregnancy-induced hypertension in regards to the number of prenatal care visits.

**Table 1:** Baseline characteristics of pregnant women and neonates according to the number of prenatal care visits*.

	The number of prenatal care visits				*P*-value
Characteristics	≤5	6–8	9–10	>10	
No.	7552(23.6%)	9904(38.9%)	8138(25.4%)	6458(20.1%)	
Maternal age (y)	25.95±3.46	25.67±3.23	25.31±2.98	25.04±2.80	<0.0001
BMI at the first pregnancy visit (kg/m^2^)	21.21±2.97	20.48±2.64	20.21±2.43	20.11±2.35	<0.0001
Gestational weight gain (kg/week)	0.54±0.23	0.54±0.18	0.53±0.17	0.52±0.15	<0.0001
Gestational age (completed week)	39(38–40)	39(38–40)	39(39–40)	40(39–41)	<0.0001
Initiation of prenatal care (week)	12(10–20)	11(9–13)	10(9–12)	10(8–12)	<0.0001
Male sex	4000(53.0%)	5162(52.1%)	4169(51.2%)	3188(49.4%)	0.0002
Pregnancy-induced hypertension^a^	149(2.0%)	230(2.4%)	152(1.9%)	141(2.2%)	0.1525
Maternal job^b^					<0.0001
Intellectuals	672(10.9%)	1118(13.3%)	1056(14.9%)	702(12.3%)	
Workers	3381(55.1%)	4971(59.2%)	4112(57.8%)	3493(61.0%)	
Farmers	1662(27.1%)	1715(20.4%)	1346(18.9%)	1049(18.3%)	
Traders	426(6.9%)	599(7.1%)	595(8.4%)	483(8.4%)	
Educational attainment^c^					<0.0001
junior middle school or below	2081(29.0%)	2392(24.5%)	1920(23.7%)	1655(25.7%)	
senior middle school	2646(36.9%)	4092(42.0%)	3525(43.6%)	2934(45.6%)	
junior college degree or above	2441(34.1%)	3270(33.5%)	2644(32.7%)	1841(28.6%)	
Prenatal care institutions^d^					<0.0001
General hospitals	6371(86.0%)	7834(80.8%)	6001(76.0%)	4414(70.0%)	
Community hospitals	1039(14.0%)	1859(19.2%)	1900(24.1%)	1889(30.0%)	

*Data are the mean ± SD, median (p25–p75) or n (%) values otherwise indicated. So is Table 2.

a missing 796, ^b^missing 4672, ^c^missing 611,^ d^missing 745.

The baseline characteristics of pregnant women and their infants according to the week of initiation of prenatal care are shown in Table 2. Approximately 71.3% of pregnant women initiated their prenatal care during the first trimester. Women with jobs as farmers were likely to have their initial care after the 12th week, whereas women with other professions tended to receive their initial care before the first trimester. In addition, women with an educational attainment level of junior middle school or below and receiving prenatal care in community hospitals were likely to have received their initial care before 9 weeks or after 12 weeks of gestation, whereas those with an educational attainment level of a junior college degree or above and receiving prenatal care in general hospitals were likely to have received their initial care during weeks 9–12 of gestation.

**Table 2:** Baseline characteristics of pregnant women and neonates according to the week of initiation of prenatal care.

	The week of initiation of prenatal care				*P*-value
Characteristics	<9	9–10	11–12	>12	
No.	6624(20.7%)	8280(25.8%)	7961(24.8%)	9187(28.7%)	
Maternal age (y)	24.94±3.17	25.67±2.85	25.69±2.96	25.65±3.52	<0.0001
BMI at the first pregnancy visit (kg/m^2^)	20.40±2.56	19.96±2.39	20.15±2.43	21.40±2.87	<0.0001
Gestational weight gain (kg/week)	0.44±0.15	0.52±0.15	0.56±0.16	0.60±0.21	<0.0001
Gestational age (completed week)	39(39–40)	39(39–40)	39(39–40)	40(39–40)	<0.0001
number of prenatal care visits (times)	9(7–10)	9(6–10)	9(6–10)	6(4–9)	<0.0001
Male sex	3489(52.7%)	4205(50.8%)	4053(50.9%)	4772(51.9%)	0.0673
Pregnancy-induced hypertension a	153(2.4%)	165(2.0%)	152(2.0%)	202(2.3%)	0.3065
Maternal job ^b^					<0.0001
Intellectuals	606(10.4%)	1200(16.2%)	1094(15.8%)	648(9.0%)	
Workers	3420(58.6%)	4453(60.2%)	4052(58.5%)	4032(55.9%)	
Farmers	1416(24.3%)	1116(15.1%)	1191(17.2%)	2049(28.4%)	
Traders	396(6.8%)	631(8.5%)	588(8.5%)	488(6.8%)	
Educational attainment ^c^					<0.0001
junior middle school or below	2301(35.2%)	1455(17.9%)	1415(18.1%)	2877(32.1%)	
senior middle school	2632(40.3%)	3401(41.9%)	3371(43.1%)	3793(42.3%)	
junior college degree or above	1606(24.6%)	3268(40.2%)	3031(38.8%)	2291(25.6%)	
Prenatal care institutions ^d^					<0.0001
General hospitals	4124(63.0%)	6789(83.8%)	6607(85.3%)	7100(79.6%)	
Community hospitals	2424(37.0%)	1309(16.2%)	1138(14.7%)	1816(20.4%)	

a missing 796, ^b^missing 4672, ^c^missing 611, ^d^missing 745.

Compared with the women with ≤5 prenatal care visits, the women who had 9–10 visits were less likely to have an LBW newborn (OR = 0.52, 95%CI: 0.38–0.71) after adjustment for the potential confounding factors. However, the protective effect was not found among the women with more than 10 visits (OR = 0.82, 95%CI: 0.57–1.18). We did not find that the late prenatal care initiation was associated with LBW risk. In addition, with the increasing of educational level, the risk of LBW among the women significantly decreased (Table 3).

**Table 3:** The LBW risk estimation associated with the prenatal care and educational level.

	Crude OR (95%CI)	Adjusted OR (95%CI)
The number of prenatal care visits^a^		
≤5	1.00 (ref.)	1.00 (ref.)
6–8	0.83 (0.69∼0.99)	0.87 (0.69∼1.11)
9–10	0.33 (0.26∼0.43)	0.52 (0.38∼0.71)
>10	0.27 (0.20∼0.36)	0.82 (0.57∼1.18)
The week of initiation of prenatal care^b^		
<9	1.00 (ref.)	1.00 (ref.)
9–10	0.92 (0.73∼1.16)	0.95 (0.71∼1.27)
11–12	0.90 (0.72∼1.14)	1.21 (0.91∼1.62)
>12	0.96 (0.77∼1.20)	1.18 (0.88∼1.60)
Educational attainment^c^		
junior middle school or below	1.00 (ref.)	1.00 (ref.)
senior middle school	0.82 (0.68∼0.99)	0.80 (0.63∼1.02)
junior college degree or above	0.71 (0.58∼0.86)	0.60 (0.46∼0.80)

abc All the three models were adjusted for maternal age, gestational weight gain, BMI at the first pregnancy visit, gestational age, pregnancy-induced hypertension (missing 796), maternal job (missing 4672), and neonatal sex, prenatal care institutions (missing 745). The week of initiation of prenatal care, the number of prenatal care visits and maternal educational attainment (missing 611) were adjusted mutually.

Hosmer-Lemeshow goodness-of-fit test for each multiple regress model: ^a^ χ^2^ = 22.99, *P* = 0.0034; ^b^ χ^2^ = 22.65, *P* = 0.0038; ^c^ χ^2^ = 20.84, *P* = 0.0076.

The joint effect between the number of prenatal care visits and the maternal educational attainment compared with the reference group on the LBW risk is presented in Table 4. After adjustment for potential confounders, generally, a decreased trend of LBW risk was found for the newborns whose mother had the higher educational level and additional prenatal care visits. And Hosmer-Lemeshow goodness-of-fit test showed that the logistic model was acceptable (χ2 = 10.46, *P* = 0.2345). There was a significant synergetic effect on the LBW risk between maternal educational attainment and the number of prenatal care visits (β = −0.1611, S_β_ = 0.0722, χ^2^ = 4.98, *P* = 0.0257), whereas no significant maternal educational attainment interaction was demonstrated with the week of initiation of prenatal care after adjusting for relevant confounding factors (β = 0.0850, S_β_ = 0.0595, χ^2^ = 2.04, *P* = 0.1530, data not shown).

**Table 4:** The LBW risk analysis associated with the number of prenatal care visits stratified by the maternal educational attainment.^#$.^

The number of prenatal care visits	Junior middle school or below		Senior middle school		Junior college degree or above	
	Crude OR (95%CI)	Adjusted OR (95%CI)	Crude OR (95%CI)	Adjusted OR (95%CI)	Crude OR (95%CI)	Adjusted OR(95%CI)
≤5	1.00 (ref.)	1.00 (ref.)	0.97(0.71∼1.33)	0.93(0.59∼1.48)	0.80(0.57∼1.12)	0.73(0.46∼1.17)
6–8	0.90(0.65∼1.26)	1.00(0.64∼1.56)	0.76(0.56∼1.02)	0.76(0.50∼1.16)	0.66(0.47∼0.91)	0.62(0.39∼0.98)
9–10	0.41(0.27∼0.64)	0.64(0.37∼1.12)	0.31(0.21∼0.46)	0.49(0.30∼0.82)	0.21(0.13∼0.35)	0.29(0.15∼0.55)
>10	0.38(0.23∼0.61)	1.33(0.74∼2.38)	0.23(0.14∼0.37)	0.67(0.38∼1.19)	0.15(0.08∼0.30)	0.37(0.16∼0.87)

# Adjusted for maternal age, gestational weight gain, BMI at the first pregnancy visit, gestational age, the week of initiation of prenatal care, number of prenatal care visits, neonatal sex, pregnancy-induced hypertension (missing 796), maternal job (missing 4672), and prenatal care institutions (missing 745). Hosmer-Lemeshow goodness-of-fit test: χ^2^ = 10.46, *P* = 0.2345.

$ The result of the interaction of the number of prenatal care visits and educational attainment on LBW risk: β = −0.1611, S_β_ = 0.0722, χ^2^ = 4.98, *P* = 0.0257.

**Figure 2:**
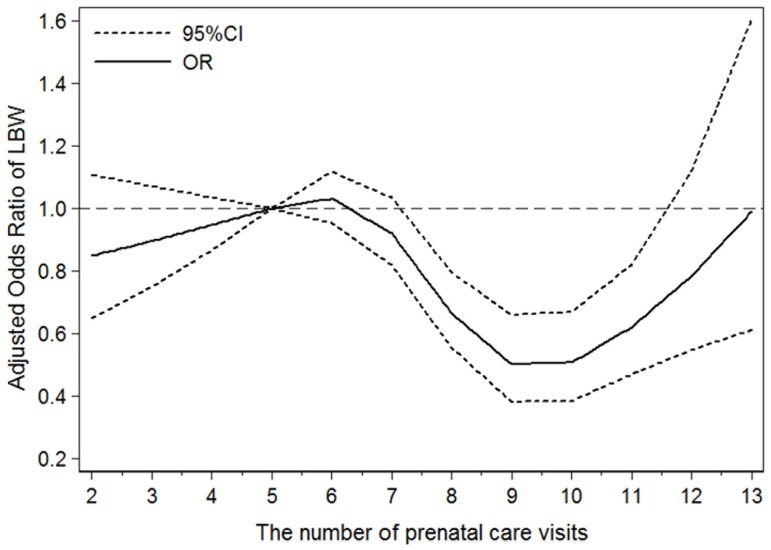
Adjusted odds ratio of LBW associated with the number of prenatal care visits. Data were fitted using a logistic model with restricted cubic splines with 4 knots (5, 7, 9, and 11 times) of the distribution of the number of visits. Estimates were adjusted for the same variables as model a in Table 3. The log likelihood value of the model was −1838.0027, and R2 was 0.2908. The probability value for nonlinearity was 0.0002.

We finally modeled the number of prenatal care visits associated with risk of LBW using restricted cubic splines (Figure 2). In total, the LBW risk displayed a ‘U-shape’ curve tendency in regards to the number of prenatal care visits (*P* for nonlinearity = 0.0002). In particular, the OR was approaching to the curve’s bottom when the women had 9 or 10 prenatal care visits. Moreover, the ORs and 95%CI of LBW risk for 7, 9, 11 and ≥13 prenatal care times were 0.92 (0.82–1.03), 0.50 (0.38–0.66), 0.62 (0.47–0.82), and 0.99 (0.61–1.60), respectively, in comparison with 5 times.

## Discussion

Two key components of this study are worth mentioning. First, the dose-response relationship analysis with restricted cubic splines shows a “U” trend between the number of prenatal care visits and the risk of LBW. Second, there is a protective interaction with decreasing the neonatal LBW risk between maternal educational level and the number of prenatal care visits but not with the week of initiation of prenatal care through drawing the dummy variables into one model.

Our data suggest that the number of prenatal care visits had a “U” trend effect on LBW risk after adjusting for a number of potential confounders, which confirms the findings of Kotelchuck [24] in 1994. In that study, prenatal care utilization was classified into 4 categories, inadequate, intermediate, adequate and adequate plus, for which the crude relative risk of LBW was 1.47, 0.56, 0.56 and 2.17, respectively. To some extent, prenatal care did have a direct protective effort on LBW risk, as demonstrated in the present study and others [6], [7], [25]. Pregnant women may enhance health awareness, abandon unhealthy habits, and get medical help through (1) early and continuing risk assessment, (2) health promotion, and (3) medical and psychosocial interventions and follow-up. Given appropriate prenatal care, fetal intrauterine growth and pregnancy complications could be detected timely and corresponding measures could be adopted by medical workers to prevent the occurrence of premature adverse obstetric outcomes.

On the other hand, our study indicates that intensive prenatal care is unable to reverse the effect on LBW. One possible explanation for this clinically plausible phenomenon is that high-risk pregnant women were more apt to receive more prenatal care with the result of being unable to avoid an LBW delivery. Researches showed that high risk factors such as low maternal educational attainment or low family income or African-American population et al., could affect pregnancy outcome regardless of the adequacy of prenatal care utilization [16], [19], [26]. And it results in those high risk pregnant women could not be able to avoid an LBW delivery successfully even if they followed doctors’ advice and attended more prenatal visits. It also indicates that the current prenatal care may not be suitable for high risk women and required reconceptualization. In our study, we suggest that women should receive 9–10 prenatal care visits in order to minimize the risk of delivering an LBW neonate. For high risk women, clinicians should pay more attention to the content and effectiveness of prenatal care other than frequent visits. However, the quality and contents of prenatal care should be considered if other researches want to confirm the results in other countries.

As proved in other studies [16], [18], [19], this study verified maternal education as an important socioeconomic factor related to the neonatal birth weight. A higher level of maternal education may be associated with higher family income and better nutrition of children, which may lead to improvement in infant birth weight. What is interesting in our study is that the maternal educational level and the number of prenatal care visits have a synergetic protective interaction on decreasing the neonatal LBW risk, which have not been previously reported. That is, compared with the baseline group, mothers with both higher educational level and a greater number of prenatal visits are more likely to avoid adverse births. One possible explanation is that pregnant women with a higher level of education more easily changed their biological, psychosocial, and behavioral factors influencing pregnancy, including poor nutrition, smoking and physical labor, than women with a lower level of education [19], [27]. In addition, education is a recognized factor affecting a person’s health awareness, attitude, and practice. Women with higher educational attainment may be more likely than other women to demonstrate health care-seeking and influence the content of their care through their requests for and adherence to provider advice on positive pregnancy-related behaviors, which may contribute to reducing their risk of LBW deliveries [28].

Prenatal care disparities due to socioeconomic inequity such as education have been reported in developed countries as well as developing countries [12], [18]. Given the increases in prenatal care resources, there is also the unavoidable phenomenon of excessive use in low-risk mothers. Super-adequate care increased from 19.5% of pregnancies in 1985 to 30.0% in 2004 in the USA, existing in every stratum defined by maternal birthplace, race, age, education, gravidity, marital status, and multiple birth [29]. In addition, randomized controlled trials have proven there is no significant difference in risk of LBW for low-risk women receiving a reduced number prenatal visits (approximately two visits fewer than recommended for existing practice) vs. those following the existing practice [30]. Some researchers have advised reinventing prenatal care as a more flexible model, with content, frequency, and timing tailored to maternal and fetal risk to improve poor birth outcomes in view of the well-intentioned but ultimately ineffectual universal prenatal care to heterogeneous groups with different medical and psychosocial risks [9]. This issue is particularly important to the government of China, a developing country with the largest population, in formulating applicable health-care policy to make full use of limited health resources to guarantee care for disadvantaged groups such as women with low educational attainment.

Three limitations of this study need to be mentioned. First, some variables (e.g., gestational age of the first or last prenatal care, maternal age at delivery, gestational age of delivery, prenatal visits and so on) have relatively high proportions of missing or implausible values. However, the variables above are similar between the original sample and the post-exclusion sample (data not shown). Second, prenatal care is multifaceted and differs between facilities and providers and contents also vary according to risk assessment or medical screening during visits. Even though we have adjusted the grade of hospitals in the multiple regression, prenatal care content in same grade hospitals could be different for all women. So the quality of prenatal care should be considered if other researches want to confirm the results in other countries. Third, the prevalence of LBW (1.86%) is relatively low, excluding stillbirth, malformation and multiple births in our study, which may limit the generalization of the findings to other areas.

### Conclusion

The finding of a “U” trend relationship between the number of prenatal care visits and the risk of LBW is useful for comprehensively understanding the effect of pregnancy care on birth weight. In addition, the finding of the effect of the protective interaction decreasing the neonatal LBW risk between maternal educational level and the number of prenatal care visits should remind medical workers to strengthen prenatal care for disadvantaged groups.

## Supporting Information

File S1 **DATA FOR PONE-D-14-10234.**
(XLS)
